# Enhancing Adherence to British Thoracic Society Guidelines for Follow-Up Chest Radiographs in Community-Acquired Pneumonia: A Quality Improvement Project

**DOI:** 10.7759/cureus.92926

**Published:** 2025-09-22

**Authors:** Aabid Nehvi, Susan Buckingham, Moein Mobini, Lauren Mills, Carolyn Meredith

**Affiliations:** 1 Emergency Medicine, East and North Hertfordshire Teaching NHS Trust, Stevenage, GBR; 2 Radiology, East and North Hertfordshire Teaching NHS Trust, Stevenage, GBR; 3 Emergency Medicine, Quality Improvement and Clinical Governance, East and North Hertfordshire Teaching NHS Trust, Stevenage, GBR

**Keywords:** bts guidelines, community-acquired pneumonia, critical alert system, emergency department, follow-up chest radiograph, nice guidelines, patient safety, quality improvement, radiology compliance, virtual pneumonia clinic

## Abstract

Background: Community-acquired pneumonia (CAP) is a common respiratory infection in adults, associated with significant morbidity and mortality, particularly among hospitalized patients. National guidelines recommend follow-up chest radiographs (CXRs) for patients with persistent symptoms or elevated risk of occult malignancy, such as smokers or those aged over 50 years. However, inconsistent adherence to these protocols, often due to process-related barriers, necessitates quality improvement initiatives to enhance patient care and reduce diagnostic delays. An internal audit identified poor compliance, prompting this quality improvement project (QIP).

Objective: To evaluate and improve adherence to British Thoracic Society/National Institute for Health and Care Excellence guidance for approximately six-week follow-up CXR after CAP using a two-cycle QIP. The primary objective was to improve CXR completion compared with baseline (32%) toward the ≥90% audit standard. Secondary objectives were to increase Virtual Pneumonia Clinic (VPC) referrals and Critical Alert System (CAS) inclusions. These aims were measurable, time-bound, and guideline-aligned, consistent with SMART framing.

Methods: A retrospective two-cycle audit in a UK ED was conducted. Cycle 1 included 50 adults with radiologically confirmed CAP (1 November 2023-3 March 2024). Outcomes were completion of a follow-up CXR at approximately six weeks, referral to a VPC (consultant-led virtual follow-up for CAP imaging/clinical review), and inclusion of CAS (electronic, closed-loop radiology alert tool for urgent/safety-critical results with mandatory acknowledgement). Although VPC and CAS are UK-specific labels, their functions, i.e., telehealth follow-up for higher-risk CAP and closed-loop radiology alerts with mandatory acknowledgement, are internationally applicable and align with American Thoracic Society/Infectious Diseases Society of America and best-practice results communication guidance. A multi-component eight-week intervention bundle was implemented (11 March-5 May 2024), comprising staff education, enhanced electronic discharge documentation with mandatory prompts, multilingual patient information leaflets, and a standardized CAS with electronic routing to the named requester. Cycle 2 re-audited 50 consecutive eligible adults (31 March-31 July 2024), and the impact of interventions was assessed.

Results: Cycle 1 showed follow-up completion of 16/50 (32%; 95% CI: 21-46), VPC referral of 12/50 (24%; 95% CI: 14-37), and CAS inclusion of 3/50 (6%; 95% CI: 2-16). Cycle 2 showed follow-up completion of 35/50 (70%; 95% CI: 56-81; χ² p<0.001), VPC referral of 38/50 (76%; 95% CI: 63-86; χ² p<0.001), and CAS inclusion of 36/50 (72%; 95% CI: 58-83; χ² p<0.001). Among ED-discharged patients who completed follow-up, ED-initiated CXR follow-up requests increased from 5/12 (42%; 95% CI: 19-68) to 24/28 (86%; 95% CI: 69-94; Fisher’s exact p=0.008). No obvious unintended effects (alert fatigue or workload escalation) were observed during the study period.

Conclusion: A low-cost, multi-component intervention produced statistically significant gains in adherence to follow-up CXR after CAP in our ED (from 16/50 (32%) to 35/50 (70%)). Embedding education, mandatory discharge prompts, patient information, and robust alerting workflows was feasible and scalable. Continued monitoring and iterative optimization are required to achieve and sustain the prespecified ≥90% compliance standard.

## Introduction

Community-acquired pneumonia (CAP) remains a frequent cause of adult morbidity and hospital admission in the United Kingdom and is associated with appreciable in-hospital mortality [1,2]. Beyond the acute episode, persistent or unresolved consolidation may conceal serious pathology, including lung cancer. Approximately 1-3% of patients initially diagnosed with pneumonia are subsequently diagnosed with malignancy [3,4]. Radiographic resolution lags clinical recovery, and about half resolve by four weeks, with most by 12 weeks [5].

United Kingdom guidance from the British Thoracic Society (BTS) and the National Institute for Health and Care Excellence (NICE) recommends arranging a follow-up chest radiograph/chest x-ray (CXR) at approximately six weeks for adults with persistent symptoms/signs after CAP or those at higher risk of underlying malignancy, particularly adults aged ≥50 years and people who smoke [1,6]. The six-week timeline was selected based on radiographic resolution kinetics [5], occult malignancy detection [3,4], and BTS/NICE guidelines [1,6], balancing timeliness and clinical utility. In parallel, novel service models such as the Virtual Pneumonia Clinic (VPC) have been piloted in the UK to deliver structured telehealth follow-up for higher-risk adults after CAP [7]. Professional guidance emphasizes closed-loop communication of imaging results (e.g., critical/urgent alert pathways and acknowledgement processes) to reduce missed or delayed follow-up [8,9], and recent National Health Service (NHS) policy promotes digital release of verified imaging reports to patients to support timely action [10]. Although “Virtual Pneumonia Clinic (VPC)” and “Critical Alert System (CAS)” are UK-specific terms, we define both in the Methods and clarify that their core functions, i.e., telehealth-based post-pneumonia follow-up for higher-risk adults and closed-loop radiology results workflows, are broadly generalizable and align with the American Thoracic Society (ATS)/Infectious Diseases Society of America (IDSA) guidance and best-practice standards for reliable communication of imaging results.

Despite clear guidance, adherence to post-pneumonia follow-up imaging remains variable across services. The BTS National Adult CAP Audit reported substantial gaps and variation in care processes, including post-discharge follow-up [11]. National data confirm that lung cancer remains one of the most common causes of cancer mortality in the UK, further underscoring the importance of timely follow-up imaging [12]. Internationally, Papavasileiou et al. [13] questioned the utility of routine follow-up for all CAP cases but endorsed targeted imaging for higher-risk groups. Similarly, Burt et al. [14] highlighted poor adherence in a UK audit, with only about half of the recommended follow-up CXRs completed. These findings reinforce that inconsistent practice remains a widespread challenge. Unwarranted variation in post-pneumonia follow-up, including missed discharge orders and incomplete closed-loop communication of urgent/critical results, heightens the risk of delayed recognition of non-resolving consolidation and missed occult malignancy, given the 4-12-week radiographic resolution window and the approximately 1-3% subsequent lung-cancer detection rate [3-5,8,9,11,12].

Our emergency department manages approximately 900-1,000 adult presentations with radiologically confirmed CAP each year, reflecting a significant local disease burden. At our institution, a preliminary review identified low compliance with recommended follow-up after CAP. We therefore undertook a two-cycle quality improvement project (QIP) to (i) evaluate baseline adherence, defined as pre-intervention performance against BTS/NICE standards on four-to-eight-week CXR completion, VPC referral, and CAS documentation, and (ii) test a pragmatic, multi-component approach to improve approximately six-week follow-up CXR in higher-risk adults. Secondary objectives were to increase referrals to a VPC and the inclusion of CAS notifications in relevant radiology reports. A prespecified local audit standard of ≥90% was set, informed by national guidance on CAP follow-up and closed-loop results management [1,6-10].

The project was previously presented at the Departmental Morbidity and Mortality Meeting and as an oral presentation at the European Congress of Radiology (ECR) 2024.

## Materials and methods

Design and setting

The hospital (catchment ~600,000; >100,000 ED attendances/year) delivered the project via a multidisciplinary team of ED clinicians (consultants, registrars, advanced clinical practitioners, nurses), radiologists (reporting/governance), respiratory physicians (VPC oversight), and administrative staff, who designed, implemented, and reviewed the project. The work followed a pragmatic PDSA (Plan-Do-Study-Act) approach. This service evaluation did not include a formal health-economic analysis (e.g., incremental cost-effectiveness ratio (ICER) and quality-adjusted life year (QALY) modeling), which was outside the predefined quality improvement (QI) scope. The intervention bundle was intentionally designed to be low-cost and operationally cost-neutral, leveraging existing electronic health record (EHR) functionality (including CAS configuration), micro-teaching embedded within routine handovers, in-house patient information materials, and existing governance structures.

Cycle 1 data collection spanned 1 November 2023 to 3 March 2024 (data abstraction/analysis: 4-10 March 2024). The intervention bundle went live on 11 March 2024 (week one), with the EHR discharge template, CAS optimization, and patient information leaflets fully operational from this date. Three interactive education sessions were delivered in weeks one, three, and six (11 March, 25 March, and 15 April). Cycle 2 accrual commenced on 31 March 2024, immediately after the week three session, ensuring that all patients were exposed to the core workflow and at least two reinforcement sessions before accrual began.

Eligibility and cohorts

Inclusion Criteria

The inclusion criteria included adults aged ≥50 years and/or current or former smokers with radiologically confirmed CAP at the index ED visit or admission (defined as community-onset lower respiratory tract infection with compatible symptoms/signs: new cough plus ≥1 of fever ≥38°C, dyspnea, pleuritic chest pain, sputum production, or focal chest findings, and a new radiographic infiltrate/consolidation on the index chest radiograph formally reported by a radiologist at ED presentation or within <48 hours of admission; cases with an alternative primary diagnosis such as isolated pulmonary edema or atelectasis were excluded).

Exclusion Criteria

Patients with known lung cancer at presentation, alternative chest imaging arranged within six weeks that superseded follow-up CXR (e.g., computed tomography), death within the follow-up window, transfer to another institution without accessible records, and hospital-onset pneumonia (≥48 h after admission) were excluded.

Cohorts

Consecutive eligible patients were included for each cycle: Cycle 1 (1 November 2023 to 3 March 2024; n=50) and Cycle 2 (31 March to 31 July 2024; n=50). Sampling was time-boxed and pragmatic, and no formal a priori power calculation was performed. Baseline adherence was defined as the pre-intervention (Cycle 1) performance, namely, the proportion of eligible CAP patients who completed a four-to-eight-week follow-up CXR, were referred to the VPC, or had CAS documentation prior to bundle implementation.

Case ascertainment and data sources

Patients were identified through systematic queries of the EHR (Cerner Millennium, Oracle Health, Kansas City, MO) and the radiology information system (RIS; PACS-Picture Archiving and Communication System) using predefined keywords (e.g., “pneumonia”, “consolidation”, “repeat in 6 weeks”, and “follow-up CXR”), and by cross-checking ED attendance logs and radiology reports for new infiltrates. For each patient, we reviewed demographics, clinical notes, discharge summaries, orders/referrals, booking/appointment records, and radiology reports. VPC referrals were verified against the clinic’s electronic booking system. For Cycle 1, follow-up completion was ascertained across the entire four-to-eight-week window after the index CXR via EHR/RIS review, ensuring complete capture despite the cohort end at 3 March.

Data abstraction and quality assurance

Two independent abstractors used a standardized codebook with preset variable definitions and decision rules. Disagreements were resolved by consensus with a third reviewer (ED consultant). Missing documentation for a given outcome (e.g., no recorded follow-up imaging) was conservatively classified as “not completed.” Data were de-identified at extraction and stored on secure Trust servers in line with institutional information-governance policies.

Outcomes and operational definitions

Primary Outcome (Process Measure)

Completion of a follow-up CXR approximately six weeks after the index CXR, operationalized as completion within 28-56 days (four to eight weeks) to reflect scheduling variability. Objectives were therefore prespecified as: (i) improve four-to-eight-week CXR completion among eligible adults, benchmarked against the institutional ≥90% standard (baseline Cycle 1 performance was subsequently observed at 32%); (ii) increase referral to VPC; and (iii) increase CAS inclusion in radiology reports. All objectives were anchored to the ≥90% audit standard, ensuring they were consistent with the SMART (specific, measurable, achievable, relevant, and time-bound) frameworks.

Secondary Outcomes

The secondary outcomes included the following: (1) referral to the VPC, i.e., a consultant-led virtual service for structured outpatient review of CAP follow-up, including CXR and clinical assessment, reducing face-to-face visits while ensuring adherence to BTS/NICE guidance [1,2,6]. The model has been implemented in several UK NHS trusts, including Southampton, where a one-year review demonstrated high follow-up rates [7] at the index encounter or during admission. (2) Inclusion of a CAS, i.e., an electronic reporting tool that delivers urgent or safety-critical radiology alerts directly to the requesting ED clinician/team inbox for acknowledgement, providing a fail-safe mechanism in line with the Royal College of Radiologists (RCR) recommendations [8], Royal College of Emergency Medicine (RCEM) standards [9], and NHS England digital notification policy [10] when follow-up was advised. (3) Source of completed follow-up initiation (ED-initiated CXR follow-up requests vs. general practice requests) among those who completed follow-up. (4) Completion of follow-up among VPC-referred patients (pathway fidelity).

Audit Standard

Before data collection, we prespecified a local performance target of ≥90% for the primary outcome (follow-up CXR completion), reflecting best-practice expectations for arranging and closing the loop on recommended follow-up.

Intervention bundle (implemented after Cycle 1)

Staff Education and Point-of-Care Prompts

Three interactive 20-30-minute handover sessions (weeks one, three, and six) led by an ED consultant with radiology liaison covered eligibility, missed-malignancy risk, and VPC/CAS workflows using short case vignettes. Reinforcement comprised laminated one-page prompt cards at workstations, posters in clinical areas, an intranet quick-reference page, and weekly email/memo reminders. Attendance was logged.

EHR Discharge Template and Order Workflow

The ED discharge template was modified to include mandatory eligibility tick-boxes and a standard phrase (e.g., “Arrange follow-up CXR at ~6 weeks and refer to VPC if age ≥50 and/or smoker or if symptoms/signs persist”). From the template, a pre-populated order (“CXR in 6 weeks - CAP follow-up”) launched with default indication text and a named responsible clinician. Hard stops prevented sign-off without a documented follow-up plan.

Patient Information and Safety-Netting

Multilingual A4 leaflets (English paired with Urdu/Polish/Romanian) were issued at discharge and auto-attached to the summary. Content explained the purpose/timing of the six-week CXR, how results would be communicated, red-flag symptoms (e.g., persistent hemoptysis and weight loss), and how to confirm or rearrange appointments.

Radiology Collaboration and CAS Optimization

Fortnightly ED and radiology meetings agreed standard CAS phrasing for CAP follow-up (e.g., “Recommend CXR at ~6 weeks to confirm resolution in patients ≥50 years, smokers, or with persistent symptoms/signs”). Alerts were routed to the requesting ED team inbox and the named clinician; acknowledgements were monitored, and a weekly exception list of unacknowledged alerts was reviewed at governance.

Governance, feedback, and fidelity checks

Weekly run-charts for key processes were displayed on the ED safety board and reviewed at monthly governance meetings. Fortnightly spot-checks of 10 random discharges audited correct template use, order placement, and leaflet provision. Immediate feedback and micro-teaching were provided when issues were identified. Minor EHR refinements were implemented iteratively during weeks seven to eight.

Balancing measures

Potential unintended consequences, such as increased staff workload or alert burden (“alert fatigue”), were monitored through brief staff feedback at handover and monthly counts of CAS alerts and acknowledgements. No changes were introduced that compromised report turnaround times or patient access to verified imaging results.

Study of the interventions

We used a pre-post (two-cycle) design without concurrent controls. Cycle 1 established baseline performance and barriers; the bundle targeted those barriers and was evaluated in Cycle 2. Cycle 2 accrued 50 consecutive eligible adults from 31 March to 31 July 2024, beginning approximately three weeks after go-live of the intervention bundle. This sequencing ensured that all patients were exposed to the core workflow (from 11 March) and that accrual commenced only after the second education session (25 March). In addition to outcome measures, we tracked fidelity (template utilization, order placement, leaflet issuance) and the balancing measures described above.

Statistical analysis

Proportions were summarized with Wilson 95% CIs. Between-cycle differences were assessed with Pearson’s chi-square test (without continuity correction). Fisher’s exact test was used when any expected cell count was <5. Crude odds ratios (ORs) with 95% CIs were calculated from 2 × 2 tables using the Wald method on the log(OR) scale. All tests were two-sided with α = 0.05. Analyses were performed in IBM SPSS Statistics version 27 (IBM Corp., Armonk, NY). Given the exploratory QI context, no adjustment for multiple comparisons was applied. Subgroup analyses (ED-discharged vs. admitted) were prespecified.

Ethics and governance

This work was registered as a service evaluation/QIP within the Trust (Trust Emergency Med_022425). Formal Research Ethics Committee review was not required; only anonymized routine data were analyzed, and no patient-identifiable results are reported.

## Results

Cohorts

Each cycle comprised 50 consecutive eligible adults with radiologically confirmed CAP (age >50 years and/or current/former smokers). In Cycle 1 (1 November 2023 to 3 March 2024), 36/50 (72%) were discharged from ED, and 14/50 (28%) were admitted. In Cycle 2 (31 March to 31 July 2024), 39/50 (78%) were discharged and 11/50 (22%) were admitted.

Primary outcomes

Follow-up CXR completion (four to eight weeks) increased from 16/50 (32%; 95% CI: 21-46) in Cycle 1 to 35/50 (70%; 95% CI: 56-81) in Cycle 2 (χ² p<0.001; absolute change +38 percentage points (pp)). Clinically, higher follow-up completion within the 4-12-week resolution window enhances detection of non-resolving consolidation and occult malignancy, enabling earlier escalation (e.g., CT or respiratory review) and reducing diagnostic delay.

VPC referral rose from 12/50 (24%; 95% CI: 14-37) to 38/50 (76%; 95% CI: 63-86) (χ² p<0.001; +52 pp). These shifts follow up from ad-hoc arrangements to a structured pathway, with active recall and symptom review, decreasing loss to follow-up and variability in care.

CAS inclusion in the index radiology report increased from 3/50 (6%; 95% CI 2-16) to 36/50 (72%; 95% CI 58-83) (χ² p<0.001; +66 pp). Embedding closed-loop alerts with mandatory acknowledgement creates a safety net against missed recommendations, strengthening the reliability of the results-communication pathway.

These gains are not only statistically significant, they represent clinically meaningful improvements in patient safety, tightening the follow-up “safety loop,” supporting earlier identification of unresolved disease, and aligning day-to-day practice with national guidance.

Table 1 compares pre- and post-intervention outcomes, demonstrating significant gains in four-to-eight-week CXR completion, VPC referral, and CAS alert inclusion.

**Table 1:** Comparative follow-up outcomes for adult CAP across two consecutive cycles (pre- vs. post-intervention) in the emergency department, with patient-level rates with 95% CIs, absolute percentage-point changes, crude ORs (95% CI), and χ²/Fisher’s test statistics. * Values are n/N (%) with Wilson 95% CIs. χ² = Pearson chi-square. Fisher’s exact test was used when expected counts were <5. Follow-up window prespecified as 28-56 days (four to eight weeks). † Denominator is ED-discharged patients who completed follow-up CXR. * Core workflow changes (mandatory EHR prompts, CAS optimization, multilingual patient leaflets) went live on 11 March 2024. Cycle 2 accrual commenced on 31 March 2024, after the week three education session, ensuring all patients were exposed to the fully operationalized intervention. Abbreviations: CAP, community-acquired pneumonia; ED, emergency department; CXR, chest radiograph; VPC, Virtual Pneumonia Clinic; CAS, Critical Alert System; CI, confidence interval; OR, odds ratio; pp, percentage points.

Outcome	Cycle 1, n/N (%)	Cycle 1, 95% CI	Cycle 2, n/N (%)	Cycle 2, 95% CI	Absolute change (pp)	Crude OR (95% CI)	χ² statistic	p-value (test)
Follow-up chest radiograph (CXR) completed at 4–8 weeks	16/50 (32%)	21–46	35/50 (70%)	56–81	+38	4.96 (2.13–11.55)	14.45	<0.001 (χ²)
Virtual Pneumonia Clinic (VPC) referral placed	12/50 (24%)	14–37	38/50 (76%)	63–86	+52	10.03 (3.98–25.27)	27.04	<0.001 (χ²)
Critical Alert System (CAS) notification included in radiology report	3/50 (6%)	2–16	36/50 (72%)	58–83	+66	40.29 (10.95–148.35)	45.78	<0.001 (χ²)
ED-initiated among ED-discharged patients who completed follow-up CXR†	5/12 (42%)	19–68	24/28 (86%)	69–94	+44	8.40 (1.94–36.37)	—	0.008 (Fisher’s)

## Discussion

This QIP demonstrated large and statistically supported improvements in adherence to recommended follow-up processes for CAP at a UK district general hospital. As detailed in Table 1, follow-up CXR completion increased from 32% to 70%, VPC referral increased from 24% to 76%, and CAS inclusion increased from 6% to 72%, with all changes being statistically significant (p<0.001). Additionally, ED-initiated follow-up requests among discharged patients who completed CXR rose from 42% to 86% (p=0.008). These findings highlight the effectiveness of embedding low-cost, pragmatic interventions directly into routine clinical workflows.

Findings in the clinical context

Follow-up imaging is critical in patients with persistent symptoms or elevated risk of occult malignancy, particularly those aged ≥50 years or with a smoking history. Approximately 1-3% of patients initially diagnosed with pneumonia are later diagnosed with lung cancer [3,4,12]. Radiographic resolution often lags behind clinical improvement; about 50% resolve at four weeks and most by 12 weeks, justifying the four-to-eight-week operational window [5]. Nationally, adherence remains poor: the BTS National Adult CAP Audit (2018/19) found that only 47.6% of survivors received a post-discharge CXR [11], comparable to our baseline of 32%. By contrast, our post-intervention rates of 70-76% exceed these benchmarks, representing a +38 pp (~2.2-fold) improvement from baseline. VPC referral rose by +52 pp (24%→76%) and CAS inclusion by +66 pp (6%→72%), demonstrating that embedding EHR defaults, hard-stops, standardized alerts, and patient-facing instructions can substantially improve closed-loop adherence. These processes are transferable to non-UK health systems using EHR portals, short message service (SMS) workflows, or telephone-based reviews. Secondary outcomes are therefore presented as process measures (pathway fidelity and closed-loop communication) rather than clinical endpoints, consistent with the QI methodology. The observed Cycle 2 outcomes (70% CXR completion, 76% VPC referral, and 72% CAS inclusion) represent measurable gains toward the ≥90% benchmark. Although incremental numeric thresholds were not prespecified, these results demonstrate how guideline-driven objectives can be operationalized in a SMART-consistent manner, providing reproducibility, transparency, and clinical relevance for international readers.

Comparison with previous quality initiatives

Our results are consistent with previously published QI interventions targeting CAP care. The Danish optiCAP (Optimizing the Management of Community-Acquired Pneumonia) project, a controlled multicenter trial, reported adherence improvements from ~20-40% to ~60-80% after tailored educational and process interventions [15]. A subsequent sustainability analysis found that without ongoing reinforcement, improvements declined over time [16]. Yealy et al. [17] demonstrated 20-30% improvements in a US emergency department trial using structured pathways and feedback. Similarly, McIntosh et al. [18] in Australia achieved increases in antibiotic guideline concordance from 25% to 55% via staff education and electronic prompts. Mortensen et al. [19] highlighted the importance of long-term follow-up due to persistent morbidity, while Waterer et al. [20] emphasized the risks of delays in CAP management. Torres et al. [21] further demonstrated the role of social determinants of health, underscoring that structural factors beyond workflow interventions may limit adherence. Dean et al. [22], in a pragmatic stepped-wedge, cluster-controlled trial, showed that real-time electronic clinical decision support improved pneumonia guideline adherence by approximately 30%, reinforcing the effectiveness of documentation-embedded interventions. Collectively, these studies demonstrate typical gains of 20-50%. Our larger improvements of 38-66 percentage points likely reflect the narrower project scope (focusing specifically on follow-up processes), the use of mandatory EHR “hard stops,” and strong multidisciplinary engagement.

Variation and Potential Harm

Process failures (absent discharge orders, lack of structured outpatient review, or non-acknowledged critical findings) are well-described antecedents of diagnostic delay, unplanned re-attendance, and worse outcomes in time-sensitive respiratory pathways. Our bundle, i.e., EHR hard-stops, a virtual review pathway (VPC), and mandatory CAS acknowledgement, directly targets these failure modes [3-5,8,9,11,12,20]. Future cycles will track patient-centered endpoints (time-to-repeat CXR/CT, 30-day re-attendance, and stage at lung cancer diagnosis) to test whether improved process reliability translates into clinical benefit.

International Applicability

Although VPC and CAS are UK-specific labels, their core functions are generalizable: telehealth-based follow-up for higher-risk adults and closed-loop radiology alerts requiring mandatory acknowledgement. These are platform-agnostic (ED- or inpatient-initiated orders; portal/SMS notifications) and align with ATS/IDSA and best-practice results-communication standards [7-10,22]. In resource-constrained settings, VPC can be delivered via nurse/physician phone reviews with pre-booked six-week CXR lists, and CAS approximated through basic electronic alerts or SMS/paper callbacks while preserving the safety loop.

Practical Benefits, Spread, and Recommendations

Beyond statistical improvements, this project delivered tangible benefits for clinicians and patients. For staff, EHR-embedded prompts and pre-populated orders reduced cognitive load and variability, clarified accountability, and streamlined closed-loop result handling; for patients, multilingual safety-netting and a coordinated follow-up pathway improved clarity and timeliness of imaging, helping to mitigate missed pathology risk. Sustained run-chart monitoring, fidelity checks, and governance feedback underpin durability and support dissemination beyond the ED (e.g., radiology and inpatient services), positioning this work as a foundation for wider organizational and regional adoption. For teams starting similar QIPs, we recommend early multidisciplinary engagement (ED, radiology, respiratory, IT, admin), operationally defined aims, maximal reuse of existing digital infrastructure (order sets, alerts, portals/SMS), brief iterative micro-teaching at routine handovers, and routine tracking of process/fidelity and balancing measures to support iterative refinement and sustainability. These pragmatic strategies are transferable across healthcare systems, supporting international adaptation and multicenter validation.

Strengths

This project incorporated prespecified outcomes (CXR completion, VPC referral, CAS inclusion) aligned with an explicit ≥90% audit standard informed by BTS, NICE, and RCR guidance [1,2,8]. Statistical robustness was ensured through chi-square and Fisher’s exact tests, with Wilson 95% CIs. Multidisciplinary collaboration between ED, radiology, and respiratory teams facilitated buy-in and sustainability. Governance monitoring and fidelity checks confirmed high intervention uptake, with 92% template utilization and 94% leaflet distribution. These factors enhanced both feasibility and reproducibility.

Limitations

Limitations of the study must be acknowledged. The single-center design restricts applicability to other institutions with different workflows and resources. The sample size (n=50 per cycle) was sufficient to detect large improvements but underpowered for rare outcomes, such as the detection of underlying malignancy. Seasonal variation (winter in Cycle 1 vs. summer in Cycle 2) and the Hawthorne effect may have influenced results. Outcomes were evaluated only up to Cycle 2, precluding conclusions on long-term sustainability or cancer detection yield. Statistical analyses were exploratory and unadjusted for multiplicity, raising the risk of type I error. Patient-level barriers such as literacy, transport, and digital engagement were not formally assessed, limiting understanding of reasons for non-compliance. Additionally, Cycle 2 accrual began on 31 March 2024, approximately three weeks after go-live of the core workflow (11 March). This ensured that all Cycle 2 patients were exposed to the operational intervention. Education continued with a final session in week six (15 April) as reinforcement only. This pragmatic sequencing is typical of QI and may conservatively underestimate the early impact of education.

The terminology used in the study is UK-specific; however, the underlying constructs are transferable to non-UK systems, warranting multicenter validation across diverse organizational and digital contexts. Finally, we did not undertake a formal health-economic analysis; the bundle was intentionally designed to be low-cost and operationally cost-neutral (leveraging existing EHR functionality, micro-teaching during routine handovers, in-house patient information, and established governance). Future work should prospectively micro-cost and model the cost per additional follow-up CXR and potential downstream savings from earlier detection/escalation, with sensitivity analyses to test robustness.

Implications and future directions

This QIP supports embedding structured follow-up processes within ED workflows. Standardized EHR prompts, patient leaflets, and CAS optimization align with national guidance on closed-loop communication from the RCR and RCEM [8,9] and complement NHS England’s digital notification policies [10]. International standards, including the ATS/IDSA guidelines, similarly recommend structured follow-up for high-risk CAP patients [23], and the NHS Right Care CAP Toolkit provides resources to support the spread and scale of such interventions [24]. To achieve the ≥90% target, sustained ED-radiology collaboration, ward-based bundles for admitted patients, and continuous governance review will be essential. Future research should evaluate cost-effectiveness, multicenter scalability, long-term malignancy detection, and patient engagement strategies such as SMS reminders or telehealth interventions. Finally, this project adhered to the Standards for Quality Improvement Reporting Excellence (SQUIRE) 2.0 reporting framework, ensuring structured reporting and transparency in quality improvement methodology [25].

## Conclusions

A multi-component intervention produced statistically significant improvements in adherence to follow-up chest radiography after CAP in our emergency department (from 16/50 (32%) to 35/50 (70%)). Embedding education, mandatory discharge prompts, patient information, and robust alerting workflows proved feasible and scalable. Importantly, the intervention was designed to be resource-neutral, utilizing existing EHR infrastructure and routine handovers, which supports its potential cost-effectiveness at a systems level. Ongoing monitoring and iterative optimization will be essential to sustain improvements and move toward the prespecified ≥90% compliance standard.
